# Decoding of novel missense *TSC2* gene variants using *in-silico* methods

**DOI:** 10.1186/s12881-019-0891-y

**Published:** 2019-10-26

**Authors:** Shruthi Sudarshan, Manoj Kumar, Punit Kaur, Atin Kumar, Sethuraman G., Savita Sapra, Sheffali Gulati, Neerja Gupta, Madhulika Kabra, Madhumita Roy Chowdhury

**Affiliations:** 1 Division of Genetics, Department of Pediatrics, AIIMS, New Delhi, India; 2 Department of Biophysics, AIIMS, New Delhi, India; 3 Department of Radiodiagnosis, AIIMS, New Delhi, India; 4 Department of Dermatology & Venerology, AIIMS, New Delhi, India; 5 Division of Neurology, Department of Pediatrics, AIIMS, New Delhi, India

**Keywords:** Tuberous sclerosis complex (TSC), Multiplex ligation dependent probe amplification (MLPA), Central nervous system (CNS), GAP (GTPase-activating protein), HGMD (human gene mutation database)

## Abstract

**Background:**

Mutations in *TSC1* or *TSC2* gene cause tuberous sclerosis complex (TSC), an autosomal dominant disorder characterized by the formation of non-malignant hamartomas in multiple vital organs. *TSC1* and *TSC2* gene products form TSC heterodimer that senses specific cell growth conditions to control mTORC1 signalling.

**Methods:**

In the present study 98 TSC patients were tested for variants in *TSC1* and *TSC2* genes and 14 novel missense variations were identified. The pathogenecity of these novel variations was determined by applying different bioinformatics tools involving computer aided protein modeling.

**Results:**

Protein modelling could be done only for ten variants which were within the functional part of the protein. Homology modeling is the most reliable method for structure prediction of a protein. Since no sequence homology structure was available for the tuberin protein, three dimensional structure was modeled by a combination of homology modeling and the predictive fold recognition and threading method using Phyre2 threading server. The best template structures for model building of the *TSC1* interacting domain, tuberin domain and GAP domain are the crystal structures of clathrin adaptor core protein, Rap1GAP catalytic domain and Ser/Thr kinase Tor protein respectively.

**Conclusions:**

In this study, an attempt has been made to assess the impact of each novel missense variant based on their *TSC1*-*TSC2* hydrophobic interactions and its effect on protein function.

## Background

Tuberous Sclerosis Complex (TSC) is a rare genetic multiorgan disorder that forms non-malignant hamartomas in brain, retina, lungs, kidneys, heart and skin. The central nervous system (CNS), skin and the renal system are most commonly affected in TSC patients. The neurological manifestations include onset of epilepsy at an infantile age, autistic features, cognitive and behavioural problems ranging from mild to severe due to the presence of structural brain abnormalities like cortical tubers, subependymal nodules (SEN), white matter lesions (WML), and subependymal giant cell astrocytomas (SEGAs) [1]. TSC is transmitted in an autosomal dominant pattern of inheritance and occurs due to mutations in one of two genes, *TSC1*and *TSC2. TSC1* gene consists of 23 exons, of which 21 encode hamartin (130 kDa), with an 8.5 kb mRNA including a 4.5 kb 3’untranslated region. The gene occupies a genomic extent of 55 kb on 9q34. The *TSC2* gene consists of 42 exons, of which 41 encode tuberin (200 kDa), with a 5.5 kb mRNA and relatively short 5′ and 3′ UTRs [2]. Both these proteins function as tumour suppressors by mediating controlled multiple cellular pathways in mammalian cells. Structurally tuberin consists of 1807 amino acids with 188 residues at carboxy terminal region having homology to the catalytic domain of Rap1/Rab5 GTPase activating proteins [3]. Tuberin plays a critical role in the regulation of cell cycle progression, differentiation and development.

Tuberin and hamartin bind together to form a GTPase activating complex (*TSC1*/2 complex) that plays a critical role in the regulation of protein synthesis, controlling cell growth and size [4]. These two proteins do not share any homology and with other proteins also very limited homology has been observed. The only evident homology that has been detected is the putative functional domain at the C-terminal of *TSC2* to the GAP (GTPase-activating protein) domain of Rap1-Gap [5]. The *TSC1*/2 exhibits GAP activity for the small GTPase, RHEB (Ras homolog enriched in brain), converting it from the active GTP bound form to the inactive state GDP bound [6]. GTP-bound RHEB promotes the kinase activity of mechanistic target of rapamycin complex1 (mTORC1), which phosphorylates a variety of downstream targets, including ribosomal S6 kinase 1 (S6K1) and eLF-4E binding protein 1 (4E-BP1) to stimulate the anabolic process like protein translation and lipid synthesis and inhibit the catabolic process such as autophagy [7]. Besides, any missense mutation in either of the *TSC1*/2 are likely to disrupt the complex formation thereby causing the disease. TSC causing mutations are identified in about 75 to 90% of the patients while in approximately10 to 15%, no pathogenic variant is identified [8]. In this study we have made an effort to ascertain the pathogenecity of novel missense variants identified in TSC patients using different bioinformatics tools and computer aided protein modeling.

## Methods

### Patient enrollment

Over a period of 5 years from 2012 to 2017, 98 patients from the age group of 0 to 16 years were enrolled from the Pediatric Neurology Clinic and Genetics Clinic of All India Institute od Medical Sciences, New Delhi. The study was approved by the institutional ethical committee and written informed consent was taken from the parents of those enrolled. Detailed clinical proforma was filled for each patients.. The diagnosis of TSC was based upon the updated clinical criteria [9] which exhibits definite and possible diagnosis based on the presence of major and minor clinical features.

### Molecular analysis for TSC

Sanger sequencing was performed on genomic DNA. Sequence analysis was performed by using ChromasPro software, NCBI BLAST and UCSC browser BLAT. For data analysis, all the variations were checked with the available human genetic variation databases online at HGMD (Human gene mutation database, http://www.hgmd.org/), dbSNP database, 1000 Genome Projects database, Ensembl browser and LOVD (http://chromium.liacs.nl/LOVD2).

### Computational assessment tools for novel missense variants

To predict the functional impact of novel missense variants, in silico prediction softwares like Polyphen [10], SIFT [11], and Mutation taster [12] were used and the splice site variant was checked with Human Splicing Finder version 2 and an improved splice site predictor tool BDGP (Berkeley Drosophila Genome Project) and Sroogle were also used to check whether this particular nucleotide change is likely to create a splice-site or not.

### Protein modelling

Three dimensional (3D) structural details of the protein help to understand their structure-function relationship at the atomic level. In the absence of an experimentally determined structure, homology modelling remains a reliable tool for the structure prediction of proteins. However the homology modelling of either domain of this protein is not possible because of the absence of any homologous structure. Hence, the second most common method to predict the 3D structure of protein, i.e. fold recognition was adopted. Threading has been used to develop the 3D model structure of the three domains (*TSC1* interacting domain, tuberin domain and GAP domain) using threading server Phyre2 [13]. Model for *TSC1* interacting domain, tuberin domain and GAP domain of human *TSC2* have been generated by combining the molecular modeling program Discovery Studio v (22) and Phyre2 server using crystal structure of clathrin adaptor core protein (PDB: 2VGL) [14], Rap1-GAP domain (PDB: 1SRQ) [15] and cryo-electron microscopy structure of Ser/Thr kinase Tor protein (PDB: 5FVM) [16] respectively. Each of these models has been energy minimized to relax the structure and remove steric constraints and verified for stereochemical quality. Model for individual variants have been built with the help of build mutant program using the optimized model structure of wild type protein. In order to analyse the structural implications of the mutant protein, the mutant protein was subjected to 20 ns molecular dynamics simulations using Gromacs Software [17].

## Results

### Identification and characterization of *TSC1/TSC2* variants

Among the total 98 TSC cases enrolled, 74 were sporadic and 24 were familial. MLPA testing for both *TSC2* gene and *TSC1* gene was done in all and results showed the presence of large genomic rearrangements in 15 out of 98 cases enrolled. Mutation analysis of the coding exons and the intron/exon junctions by Sanger sequencing was carried out in the remaining 85 TSC cases.

Among the total 85 TSC cases, 71 different variants were identified in either *TSC1* and *TSC2* gene in 78 cases, while no mutation was found in 7 cases. Out of 71 variants, 64 were found in *TSC2* gene while remaining 7 of these in *TSC1* gene. The 7 TSC1 variants included 3 nonsense, 1 splice-site, 1 small deletion and 2 small insertion variants, while the 64 TSC2 variants included 23 missense, 17 splice-site, 11 nonsense, 9 small deletion, and 4 small insertion variants.

Out of the total 23 missense variants, 9 missense variants are already reported as disease causing variants while the remaining 14 likely pathogenic variants were first checked in parents and siblings and in the multiple databases which included LOVD, Ensemble, HGMD, 1000 genome and also in different repository databases of Indian population as well as from other countries before these variants were marked as novel. List of all 14 novel missense variants in *TSC2* gene is shown in Table 1.

**Table 1:** List of 14 novel missense *TSC2* mutations

S.No.	Patient ID	Inheritance	*TSC2* Exon Number	Mutation Site	Amino acid alteration	Domain-wise distribution in tuberin
1	TS-116	Sporadic	20	c.2150 T > C	p.Leu717Pro	Domain 2
2	TS-7	**Familial**	34	c.4490C > T	p.Pro1497Leu	Domain 3
3	TS-9	Sporadic	6	c.581A > T	p.Tyr194Phe	Domain 1
4	TS-97	Sporadic	18	c.1930 T > G	p.Cys644Gly	Domain 2
5	TS-59	Sporadic	14	c.1516A > G	p.Lys506Glu	Domain 2
6	TS-101	Sporadic	38	c.4859A > C	p.His1620Pro	Domain 3
7	TS-112	**Familial**	39	c.5018 T > C	p.Val1673Ala	Domain 3
8	TS-67	Sporadic	11	c.1058 T > C	p.Leu353Pro	Domain 1
9	TS-35	Sporadic	16	c.1835 T > C	p.Leu612Pro	Domain 2
10	TS-54	Sporadic	16	c.1636G > A	p.Glu546Lys	Domain 2
11	TS-42	**Familial**	13	c.1361G > A	p.Arg454Lys	Domain 1
12	TS-43	Sporadic	31	c.3713C > T	p.Ala1238Val	Domain 3
13	TS-48	Sporadic	10	c.896 T > A	p.Val299Glu	Domain 1
14	TS-32	Sporadic	31	c.3715G > A	p.Glu1230Lys	Domain 3

Boldface is used to differentiate familial case from sporadic case

**Fig. 1:**
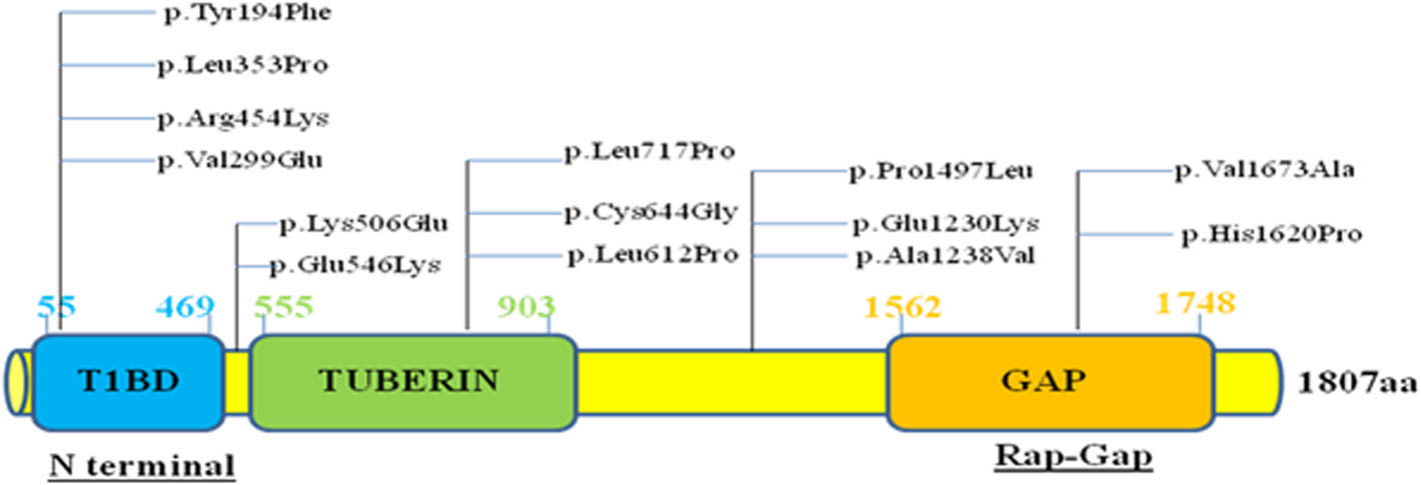
Schematic representation of 13 novel *TSC2* missense variants identified in respective domains of *TSC2* protein

Of the 14 observed novel missense variants found in tuberin protein, 10 observed variants (**Tyr194Phe, Val299Glu, Leu353Pro, Lys506Glu, Glu546Lys, Leu612Pro, Cys644Gly, Leu717Pro, Pro1497Leu and Val1673Ala**) lie in the defined regions of N terminal *TSC1* interacting region (domain 1), tuberin (domain 2) and GAP (domain 3) respectively and only those could be modelled to study their effect on the structure of the protein. Figure 1 represents domain structure of *TSC2* protein and also specifies the position of each novel missense variants in the respective domains.

### Structural characterization for novel missense variants identified in *TSC2* gene

Human TSC2 protein comprises of 1807 residues, and acts as a tumour suppressor in complex with *TSC1*. Three regions, N terminal *TSC1* interacting region (residues 55 to 469), tuberin type domain (residues 555 to 903) and GTPase activator (GAP) domain (residues 1562 to 1748) are distinct on the basis of sequence similarity search with protein domain families. The pathogenecity of the various variants observed was assessed by comparing the modelled three-dimensional structures of the wild type protein with the respective individual mutant structures. The variants were categorized based on their presence in specific domain regions.

#### Domain 1 (N terminal *TSC1* interacting region): the N terminal *TSC1* interacting region in tuberin protein comprises of alpha-alpha superhelix domain

This domain is stabilized by hydrophobic interactions between each helix.

**Fig. 2:**
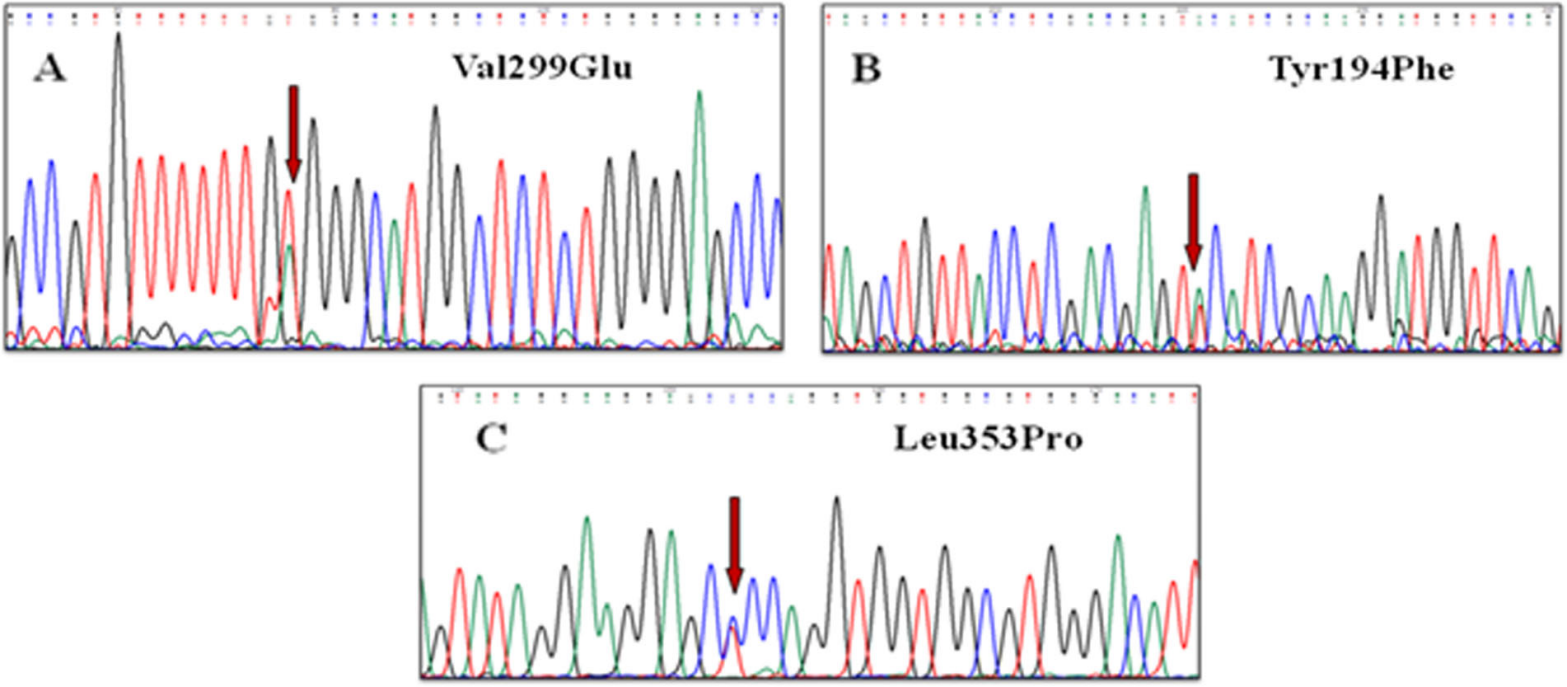
Electropherogram showing variants identified in Domain 1, (**a**) Val299Glu (**b**) Tyr194Phe (**c**) Leu353Pro, of tuberin protein

Four variants were found in this domain (p.Tyr194Phe, p.Leu353Pro, p.Arg454Lys, and p.Val299Glu. Protein modeling was done for Tyr194Phe, Val299Glu, and Leu353Pro (Fig. 2). For p.Arg454Lys, no homology protein sequence could be identified to generate model structure.

**Fig. 3:**
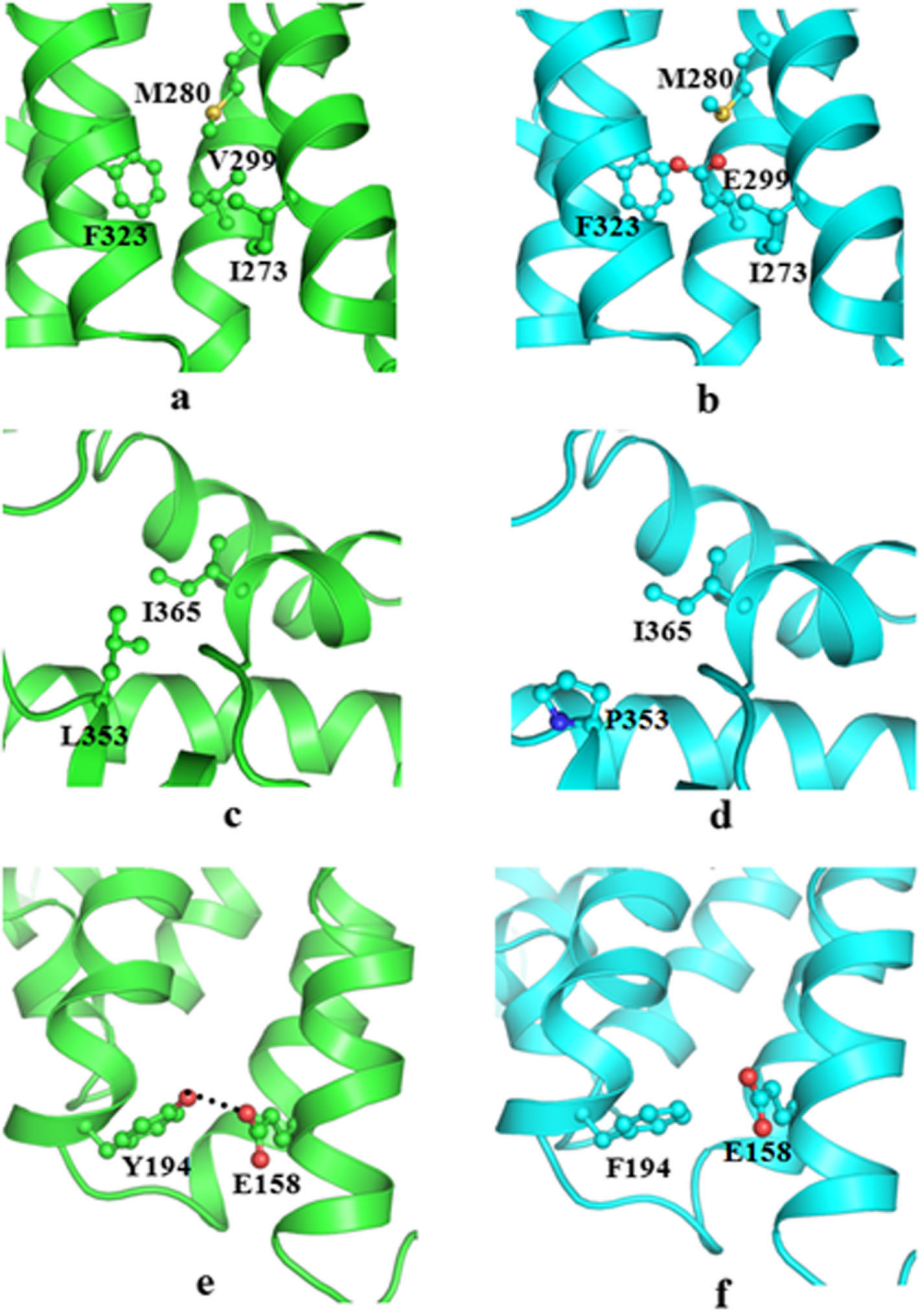
Cartoon representation of *TSC1* interacting domain of wild type*TSC2* (cartoon, green) in (**a**, **c** & **e**) and mutants (cartoon, cyan) in (**b**) Val299Glu, (**d**) Leu353Pro and (**f**) Tyr194Phe. Important residues have been represented in ball and stick and hydrogen a bonded interaction is shown as black dotted lines

The TSC1 interacting region in tuberin protein (residues 55 to 469) comprises an alpha-alpha superhelix domain encompasses the variants involving residues Tyr194, Val299 and Leu353. The hydrophobic residue Val299 located in the middle of the long helix (Fig. 3a) forms van der Waals contacts with three other hydrophobic residues Ile273, Met280 and Phe323 occurring on neighbouring helices. These interactions contribute to stabilize the helix bundle of alpha-alpha superhelix domain. The substitution of hydrophobic residue Val299 by a highly polar and negatively charged residue Glu299 (Fig. 3) not only disrupts the hydrophobic interactions between helices, but also changes the non-polar environment of the buried area in the domain into negatively charged polar environment (Fig. 3b). This might destabilize the alpha-alpha helix bundle and affect the interaction with *TSC1*.

In case of L353P variant, the hydrophobic residue Leu353 lies at the beginning of the helix and is stabilized through interactions with the neighbouring hydrophobic residue Ile365. This hydrophobic interaction contributes in keeping the motif intact (Fig. 3c). Substitution of Leu353 in mutant protein by the imino group containing Pro353 (Fig. 3) disrupts the helix at this position and substantially weakens the hydrophobic interaction with respect to wild type protein (Fig. 3d). As a result it most likely adversely affects the functioning of this protein.

In the wild structure, the hydroxyl group of Tyr194 side chain interacts with the side chain of Glu158 through hydrogen bonds which contribute to stabilize the helix interfaces (Fig. 3e). The substitute Phe in case of Tyr194Phe mutant though like Tyr is an aromatic residue but lacks the hydroxyl group present on the Tyr side chain. This results in the loss of the hydrogen bond which in turn weakens the interaction between two helices and decreases the overall stability of the domain.

##### Clinical assessment

The detailed clinical assessment for all 4 TSC patients showed presence of early onset of seizures and presence of brain lesions in the form of cortical tubers, SENs and WMLs. The dermatological features included only presence of ash leaf spots as all patients were below 5 years of age.

#### Domain 2: tuberin domain

The tuberin domain of TSC2 protein comprises mainly alpha helices connected by loops in an arrangement similar to alpha-alpha superhelix domain and consists of two distinct subdomains linked through a long helix. The substitutions observed involved residues Lys506 and Glu546 in subdomain 1 and residues Cys644 and Leu717 in subdomain 2.

Five variants were identified in the tuberin domain (p.Lys506Glu, p.Glu546Lys, p.Leu717Pro, p.Cys644Gly, and p.Leu612Pro). Protein modeling was done for Lys506Glu and Glu546Lys present in subdomain 1 and residues Cys644Gly and Leu717Pro in subdomain 2.

**Fig. 4:**
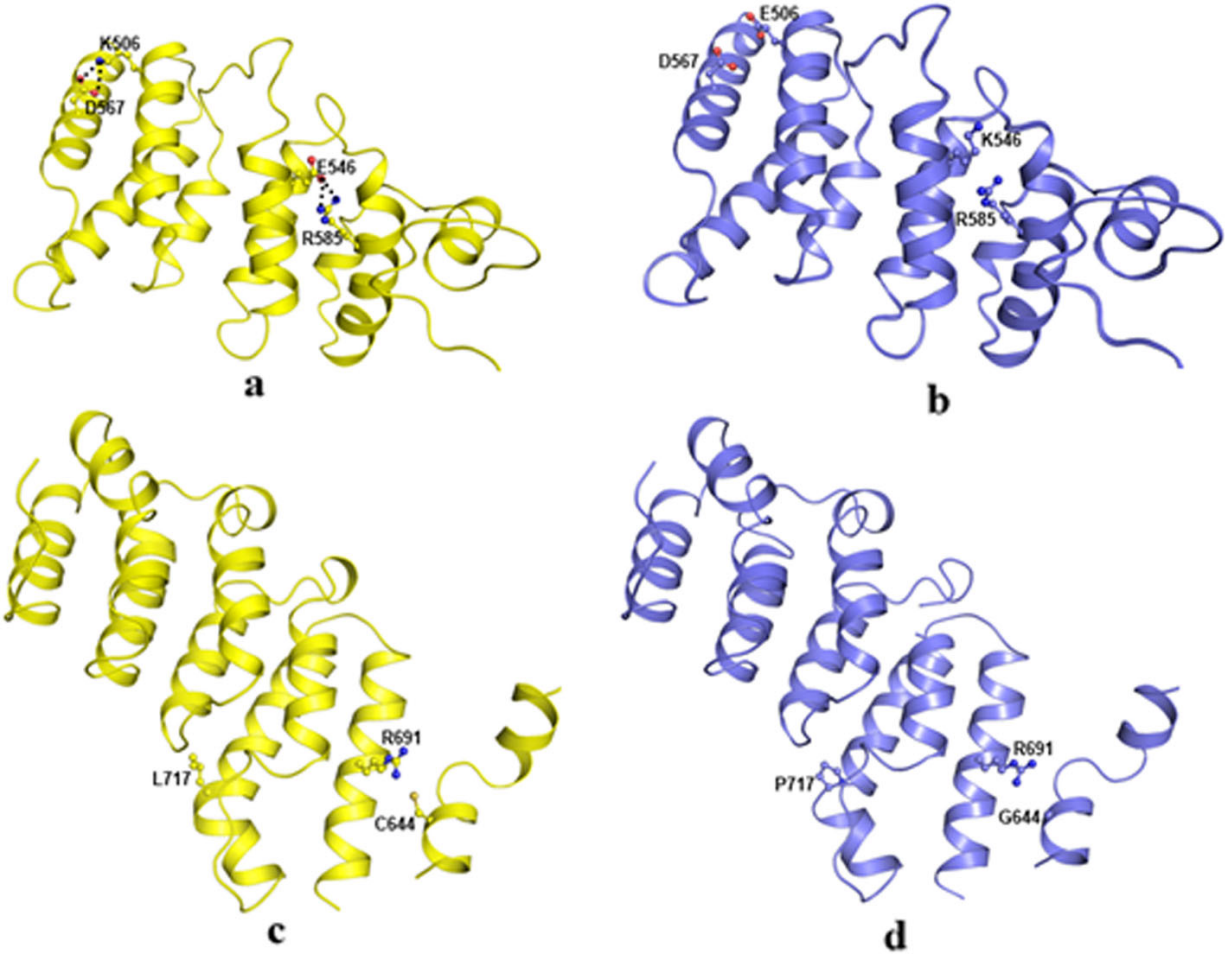
Cartoon representation of tuberin domain of wild type *TSC2* (cartoon, yellow) in (**a** & **c**) and (**b**) mutants (cartoon, blue) in (**b**) Lys506Glu and (**d**) Glu546Lys. Important residues have been represented in ball and stick and hydrogen bonded interactions are shown as black dotted lines

In the wild type protein, the positively charged residue Lys506 present in subdomain 1 on the alpha helix forms a salt bridge with negatively charged side chain of Asp567 present on neighbouring helix (Fig. 4a). This is the most significant interaction between these two helices. Replacement of positively charged Lys506 in the mutant with the negatively charged Glu506 (Fig. 4) disturbs the charge, environment leading to the loss of the salt bridge and consequently repels the similarly charged side chain of Asp567 (Fig. 4b). This will disturb the interaction between the two helices and adversely impact the stability of the structure.

The presence of the oppositely charged residues, negatively charged Glu546 and positively charged Arg585 on the neighbouring helices results in the formation of a salt bridge which stabilizes the helix-helix interaction (Fig. 4a). The substitution of Glu546 by a positively charged residue Lys546 (Fig. 4) alters the polar environment and pushes the similarly charged Arg585 away (Fig. 4b). The variant resulting in the loss of salt bridge will adversely impact the helix-helix interaction and might consequently destroy the protein conformation necessary for the dimerization of the protein.

Subdomain 2 consists predominantly of alpha helices similar to the subdomain 1. Residue Cys644 in the wild type protein resides on the linker helix. Due to the absence of an another cysteine residue in the vicinity, it forms a weak hydrogen bond with Arg691 present on the first helix of the second subdomain (Fig. 4c). The mutant has Gly in place of Cys644 which is a supple residue and lacks a side chain. Hence the interaction between subdomain 1 and linker helix is lost in the mutant (Fig. 4d). Another mutant concerning Leu717 residue is located in the long helix in subdomain 2 (Fig. 4c). In the mutant this is replaced by smaller Pro residue. Pro residue contains an imino group and is a well known helix breaker. The replacement of Leu by the Pro in mutant protein unwinds the helix and alters the backbone structure locally (Fig. 4d). This might be a destabilizing factor for the protein.

##### Clinical assessment

The detailed clinical assessment for these 4 TSC patients showed no specific phenotype.

#### Domain 3: Rap-Gap domain

The model structure of GAP domain of *TSC2* shows the presence of beta sheet consisting of seven parallel beta strands surrounded by alpha helices forming an alpha-beta fold. This domain has a catalytic role of GTP hydrolysis. The observed variant Pro1497 in this domain lies in the loop region and connects this domain to another domain of the *TSC2* protein (Fig. 6a).

**Fig. 5:**
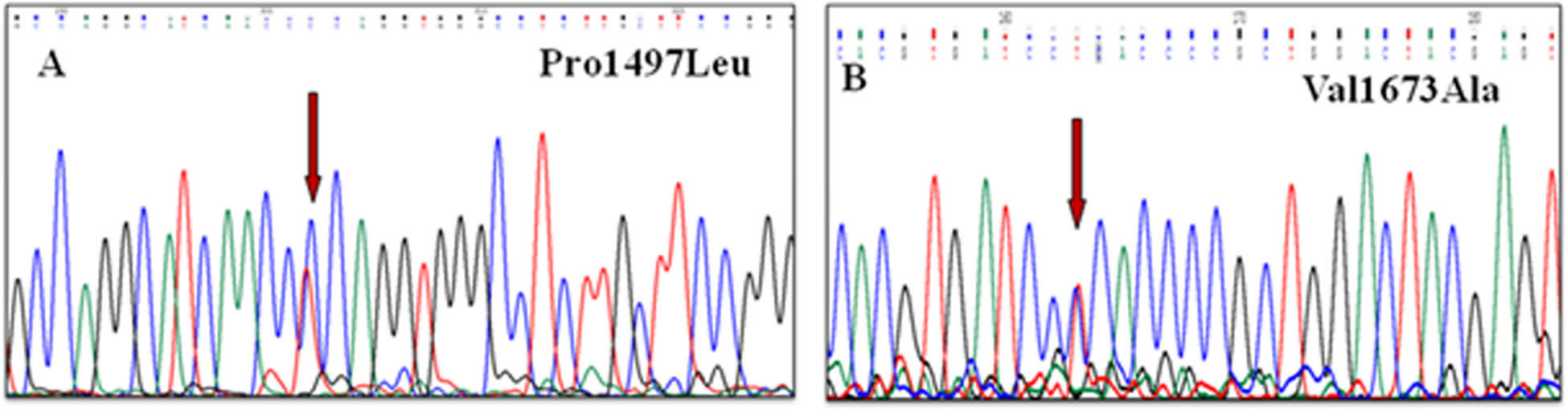
Electropherogram showing variants identified in Domain 3, (**a**) Pro1497Leu (**b**) Val1673Ala, of tuberin protein

Five variants (p.Pro1497Leu, p.Glu1230Lys, p.Ala1238Val, p.Val1673Ala, and p.His1620Pro) were found in Rap-Gap domain. Protein modeling was done for Pro1497Leu, and Val1673Ala (Fig. 5). The other variants could not be modeled due to the lack of a suitable homology sequence.

**Fig. 6:**
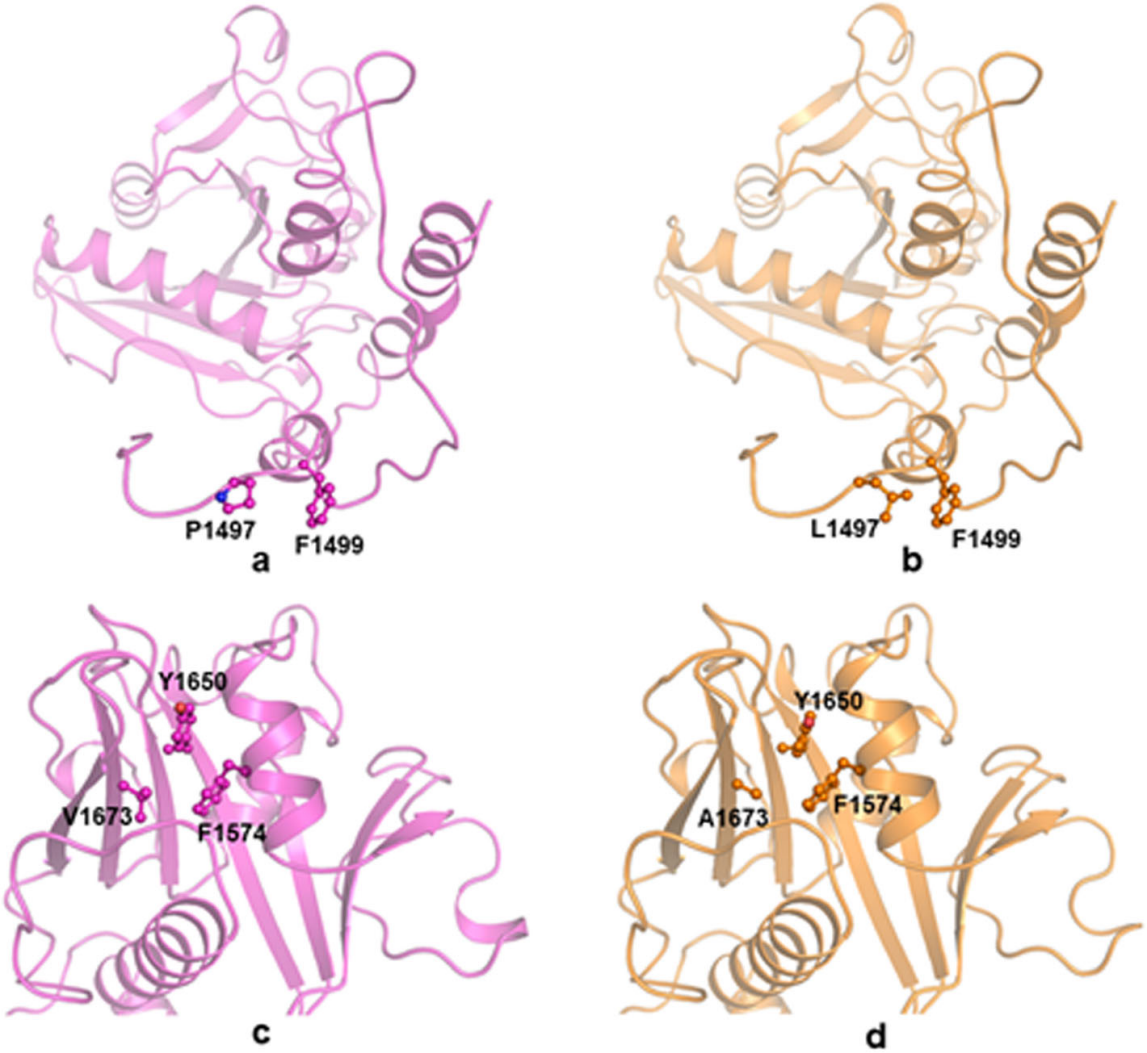
Cartoon representation of GTPase (GAP) domain of wild type (magenta) TSC2 in (**a** and **c**) and mutants (orange) in (**b**) Pro1497Leu and (**d**) Val1673Ala. Important residues have been represented in ball and stick

The imino group containing Pro residue is also known to provide rigidity in protein structure due to its restricted geometry which could be crucial for the proper arrangement of domains and hence the domain-domain interactions for the functional integrity of *TSC2* protein. The substituted residue Leu (Fig. 6) is also a non-polar residue but lacks the rigidity provided by Pro residue. Though Leu1497 can interact with non-polar residue Phe1499 in the same loop through hydrophobic interaction, but it fails to provide the required rigidity and conformation imparted by Pro. The lack of restricted movement (provided by Pro in the wild type) and resultant greater flexibility of the loop due to presence of Leu (in the mutant) may adversely affect the interaction between the two flanking domains due to possibly greater inter-domain movement resulting in diminished function of the protein.

The Val1673Ala variant might adversely impact the function (Fig. 6). Val1673 is a non-polar residue packed in the hydrophobic pocket surrounded by Phe1574 and Tyr1650. The substituted Ala is smaller in size and found to be less hydrophobic than Val and this substitution results in the loss of hydrophobic interactions. This may affect the packing and hydrophobic arrangement in the region and hinder GAP activity.

##### Clinical assessment

The detailed clinical assessment for all patients showed presence of seizures and brain lesions. All patients had milder form of neurocognitive outcome as compared to those having variants in domain 1 & 2.

## Discussion

Studies indicate that the impact of the missense change depends on criteria such as the evolutionary conservation of an amino acid or nucleotide, the location and context within the protein sequence, and the biochemical consequences of the amino acid substitution. As per the study by Thusberg et al*.* [18] most of the prediction algorithms used for the prediction of missense variants are 65–80% accurate when examining known disease variants. As per the ACMG standards and guidelines, it recommends that multiple prediction software programs should be used for sequence variant interpretation as each in silico tools have their own weaknesses and strengths.

There were 23 novel variants identified in the present study, of which 14 were missense variants, 3 were inherited variants while remaining 11 were de novo origin as shown in Table 1. These variants were checked in 1000 Genome, dbSNP, HGMD, LOVD and different repository databases of commercial companies like Centogene, MedGenome and Strands and none of these were found in any ethnic population.

Molecular modeling was done for 10 of the total 14 missense variants as for remaining 4 variants no homology protein sequence was identified to generate model structure. The 10 variants were found disrupting the hydrophobic interactions between helices and were also found changing the polarity of the affected domains. This destabilizes the protein structure, thereby affecting the interaction with *TSC1*. The three dimensional (3D) model of tuberin protein domains was constructed by using a combination of threading server Phyre2 and Discovery studio v2.5 molecular modeling program. The known crystal structure of clathrin adaptor core protein, Rap1-GAP domain and cryo-electron microscopy structure of Ser/Thr kinase Tor protein was used as template respectively. The initial models of both the wild type and mutant type of tuberin protein were subsequently subjected to molecular dynamics (MD) simulations to relax the structure by energy minimization and were verified for stereochemical quality.

The three variants were identified in domain 1 of tuberin protein, which included p.Tyr194Phe, p.Val299Glu and p.Leu353Pro. The substitution at 299 position disrupted the hydrophobic interactions and also changed the polarity of helices from non polar to negatively charged helices. Prolines are known for their rigid structure conformation while at times it forces to change the backbone of protein structure into specific conformation. The substitution at 353 position has disrupted the hydrophobic interactions which weakened the helices and had changed the structure conformation of the protein. Another substitution at 194 position has modified the stability of helices by loss of hydrogen bond interaction thereby decreasing the stability of the domain as shown in Fig. 2.

Similarly in domain 2, among the 5 variants identified protein modeling was done for 4 variants. The substitution at 506 position adversely affected the stability of the structure by disrupting the most significant salt bridge interaction between two helices. Another substitution at 546 by a positively charged residue has changed the polarity of the helices. The substitution at 644 and 717 position weakens the hydrogen bond in helices and the proline in the mutant structure unwinds the helices thereby resulting in disrupting the domain structure. These variants have an adverse impact on the helix-helix interaction and might have destroyed the protein conformation which is necessary for the dimerization of the protein.

The Rap-GAP domain had five variants identified and protein modeling was done for two of them. The substitution at 1497 position disrupted the rigidity of the protein conformation by affecting the stability of the loop in the helical region. Another substitution at 1673 position disrupted the hydrophobic interactions within the helical region which might have affected the packing and hydrophobic arrangement in the region and hindered GAP activity.

Studies from different populations have provided insights about the functional relevance of tuberin and hamartin proteins stating that it might affect cell proliferation, growth, adhesion, migration or protein trafficking [5]. The C- terminal coiled coil domain of hamartin protein is necessary for the interaction with tuberin protein directly to form a complex structure. Also tuberin gets phosphorylated at its serine and tyrosine residues which also affects the interaction between the two proteins to form complex [19]. The GAP domain is responsible for the inhibition of cell division by indirectly modulating the mTOR which is a key regulator of translation [20].

## Conclusion

It is always a challenging task to predict the effect of missense variants on protein function. Analyzing familial segregation could help but the small family size and lack of familial clinical information in simplex cases make the segregation analysis challenging. Considering the functionality of these domains, it is very likely that these missense variants are directly affecting by interfering in protein folding, their charge and hydrophobic interactions leading to protein truncation. To determine the pathogenecity of novel variants many alternative protocols to animal studies are being used nowadays which can provide dependable outcomes. To ascertain whether the novel variants identified in TSC patients are disease causing or benign, an attempt was made to understand the level and extent of pathogenecity of these identified novel variants by using different bioinformatics tools and computer aided protein modeling methods.

## Data Availability

The protein modeling data sets generated during the current study are available from the authors from Department of Biophysics on request. Dr. Punit Kaur: punitkaur1@hotmail.com Dr. Manoj Kumar: manmath.manoj@gmail.com

## Abbreviations

CNS: Central nervous system
RHEB: Ras homolog enriched in brain
SEGAs: Subependymal giant cell astrocytomas
SEN: Subependymal nodules
TSC: Tuberous Sclerosis Complex
WML: White matter lesions
